# Differential Regulation of Lacto-/Neolacto- Glycosphingolipid Biosynthesis Pathway Reveals Transcription Factors as Potential Candidates in Triple-Negative Breast Cancer

**DOI:** 10.3390/cancers13133330

**Published:** 2021-07-02

**Authors:** Ruichao Zeng, Ahmed Mohamed, Kum Kum Khanna, Michelle M. Hill

**Affiliations:** 1 Precision & Systems Biomedicine Laboratory, QIMR Berghofer Medical Research Institute, Brisbane 4006, Australia; ruichao.zeng@remegen.cn (R.Z.); mohamed.a@wehi.edu.au (A.M.); 2 Signal Transduction Laboratory, QIMR Berghofer Medical Research Institute, Brisbane 4006, Australia; kumkum.khanna@qimrberghofer.edu.au; 3 University of Queensland Centre for Clinical Research, Faculty of Medicine, The University of Queensland, Brisbane 4006, Australia

**Keywords:** triple-negative breast cancer, glycosylation, glycogenes, glycosphingolipids, lacto, neo-lacto, transcription factor

## Abstract

**Simple Summary:**

Triple-negative breast cancer (TNBC) tends to occur in younger women, is aggressive and has a poor outcome due to limited therapies. Recent drug trials have shown promise, but only improved TNBC survival by several months. To discover potential new therapeutic targets and pathways, we focused on the known alteration of glycosylation in cancer, and sought to discover TNBC-specific glycogenes and their regulatory pathways. Using an integrative bioinformatics approach, we discovered 34 TNBC-specific candidate glycogenes, and identified the lacto-/neolacto- glycosphingolipid biosynthesis pathway with seven candidate glycogenes as a novel target in TNBC. Furthermore, we identified three transcription factors as potential therapeutic targets: AR, GATA3 and ZNF622. Each TF target three glycogenes in this pathway. Together, this study revealed novel molecular features of TNBC, identifying potential new therapeutic targets.

**Abstract:**

Triple-negative breast cancer (TNBC) is an aggressive breast cancer with limited treatment options. Glycosylation has been implicated in cancer development, but TNBC-specific glycosylation pathways have not been examined. Here, we applied bioinformatic analyses on public datasets to discover TNBC-specific glycogenes and pathways, as well as their upstream regulatory mechanisms. Unsupervised clustering of 345 glycogene expressions in breast cancer datasets revealed a relative homogenous expression pattern in basal-like TNBC subtype. Differential expression analyses of the 345 glycogenes between basal-like TNBC (hereafter termed TNBC) and other BC subtypes, or normal controls, revealed 84 differential glycogenes in TNBC. Pathway enrichment showed two common TNBC-enriched pathways across all three datasets, cell cycle and lacto-/neolacto- glycosphingolipid (GSL) biosynthesis, while a total of four glycosylation-related pathways were significantly enriched in TNBC. We applied a selection criterion of the top 50% differential anabolic/catabolic glycogenes in the enriched pathways to define 34 TNBC-specific glycogenes. The lacto-/neolacto- GSL biosynthesis pathway was the most highly enriched, with seven glycogenes all up-regulated in TNBC. This data led us to investigate the hypothesis that a common upstream mechanism in TNBC up-regulates the lacto-/neolacto-GSL biosynthesis pathway. Using public multi-omic datasets, we excluded the involvement of copy-number alteration and DNA methylation, but identified three transcription factors (AR, GATA3 and ZNG622) that each target three candidate genes in the lacto-/neolacto- GSL biosynthesis pathway. Interestingly, a subset of TNBC has been reported to express AR and GATA3, and AR antagonists are being trialed for TNBC. Our findings suggest that AR and GATA3 may contribute to TNBC via GSL regulation, and provide a list of candidate glycogenes for further investigation.

## 1. Introduction

Breast cancer (BC) is the most common cancer and leading cause of cancer death worldwide in females [1]. Currently, BC treatment choice is based on the hormone receptor expression of the tumour, with hormone- and receptor-targeted therapies achieving long-term outcomes for oestrogen receptor-positive (ER+), progesterone receptor-positive (PR+) and/or human epidermal growth factor receptor-positive (HER2+) BC [2]. However, approximately 15% of BC cases lack ER, PR and HER2 expression (termed triple-negative breast cancer, TNBC), often occurring in premenopausal women and displaying aggressive features [3]. Compared with other BC subtypes, TNBC has the worst prognosis, with a 40% mortality rate within the first 5 years after diagnosis [4]. 

With the lack of hormone- or receptor-targeted therapies, TNBC is generally treated by surgery and postoperative chemotherapy with limited efficacy, which results in tumour progression and relapse [2]. The average time to relapse is significantly higher in non-TNBC than that in TNBC, 35–67 months vs. 19–40 months [4]. TNBC with residual disease after neoadjuvant chemotherapy can rapidly progress within 3 to 5 years, and TNBC with relapse showed less than 1-year median overall survival [5,6,7]. Thus, there is an urgent need to understand the oncogenic mechanisms underlying TNBC, and to develop early diagnostic biomarkers and novel therapeutic targets for TNBC. 

To uncover molecular pathways and novel therapeutic targets, intrinsic BC subtyping methods were investigated using gene expression profiles. The PAM50 classifier has been widely used and classifies BC into five subtypes based on expression of 50 genes, namely luminal A, luminal B, HER2-enriched, basal-like (BL), and normal-like [8,9,10,11]. The majority (~70%) of TNBCs are classified as the BL subtype, with DNA damage response identified as a target pathway [12,13,14,15]. DNA repair-pathway-targeted therapies, such as PARP inhibitors, have been shown to improve progression-free survival in TNBCs with germline BRCA mutations in phase II and III trials, however overall survival benefit was not achieved [16]. More recently, immunotherapy targeting the anti-PD1/PD-L1 axis has shown efficacy in ~20% of TNBCs; however, biomarkers are needed to identify which patients are not likely to respond, and others which might have a durable response [17,18]. As a complementary approach for novel therapeutic target discovery, we sought to identify TNBC-enriched glycosylation-related genes (glycogenes) and their regulatory pathways, as altered glycosylation has been widely implicated in cancers. 

Glycosylation is a common post-translation modification involving the enzymatic transfer of glycans to proteins or lipids [19]. The highly complex cellular glycome can be broadly divided into N-linked, O-linked glycoproteins, glycolipids and proteoglycans [8]. Glycoconjugates comprised of different saccharides in a variety of linkages generate a diversity of glycoforms for a single protein/lipid, which leads to the fine-tuning of its structure and binding partners, potentially impacting functions. The type and level of glycosylation is dependent on multiple factors, including the expression and location of glycosylation biosynthetic and catabolic enzymes, and the availability of glycan precursors.

Altered glycosylation has been implicated in multiple cancer hallmarks to impact cancer development, progression and metastasis [20]. Furthermore, differential glycosylation of target molecules, such as immune checkpoint PD-1/PD-L1, influences therapeutic responses [21,22]; therefore, glycosylation has emerged as a new frontier in cancer and personalized therapies. The potential importance of glycosylation in TNBC therapy is illustrated by the equivalent effect of a therapeutic antibody on glycosylated forms of PD-L1 (with poly-N-acetyllactosamine on N192 and N200) in a TNBC mouse model and downregulation of β-1,3-N-acetylglucosaminyl transferase (B3GNT3) in TNBC cells [22]. While differential glycogene expression has been implicated in various cancer types, including BC [8,23] and basal-like BC [12,15], it is not known whether TNBC exhibits unique glycogene expression signatures which could be used as potential biomarker and therapeutic targets. 

To discover TNBC-specific glycosylation genes, we interrogated three public breast cancer gene expression datasets with a pre-defined list of 345 glycogenes. Through unsupervised hierarchical clustering, we found that TNBC exhibits a unique glycogene expression profile compared to normal-like and other BC subtypes. Inspection of molecular subtypes of TNBC revealed clustering of the BL subtype. Focusing on BL TNBC, we identified 84 differentially expressed glycogenes and four enriched glycosylation pathways that were consistent across the three datasets. Filtering for four pre-defined criteria generated a list of 34 candidate glycogenes for TNBC and identified the lacto-/neolacto glycosphingolipid (GSL) biosynthesis pathway to be activated in TNBC. We went on to investigate upstream regulatory mechanisms associated with the activation of this pathway in TNBC, discovering transcription factors (TFs) that regulate multiple glycogenes in this pathway. Overall, these results provide new mechanistic insights in TNBC, as well as novel candidates for targeted therapy development. 

## 2. Materials and Methods

### 2.1. Study Design

**Figure 1:**
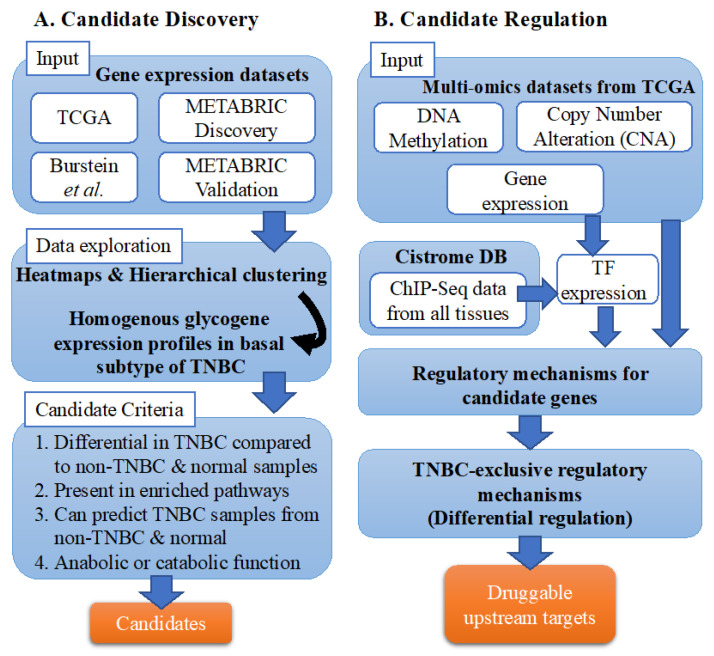
Study design and workflow. The study comprised two phases, discovery of candidate TNBC glycogenes (**A**), and identification of upstream regulatory mechanisms (**B**). TNBC, triple-negative breast cancer; TF, transcription factor.

The study comprised two phases, candidate discovery and identification of upstream regulatory mechanisms (Figure 1).

### 2.2. Data Sources and Processing

**Figure 2:**
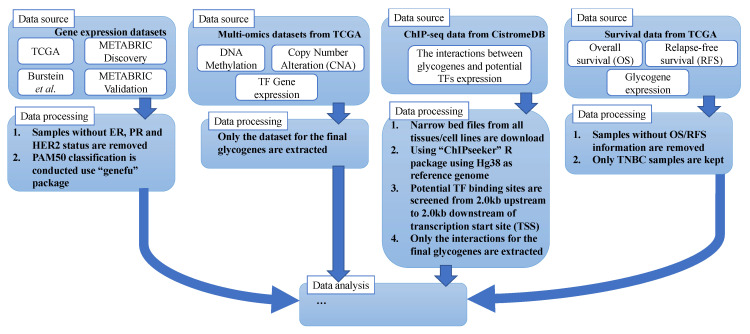
Data sources and processing summary.

Data sources and processing methods are summarized in Figure 2. Gene expression datasets were downloaded from three sources: (1) The Cancer Genome Atlas (TCGA) dataset, with clinical information obtained from UCSC Xena (https://xenabrowser.net/datapages/, accessed on 1 February 2017) [24,25]; (2) Molecular Taxonomy of Breast Cancer International Consortium (METABRIC) Discovery and Validation datasets with clinical information (https://ega-archive.org/studies/EGAS00000000083, accessed on 1 March 2017) [26]; and (3) Burstein’s dataset attained by “GEOquery” package from GSE76275, comprising 198 TNBC samples [27]. The available ER, PR and HER2 status of the samples were used to classify samples in TCGA and METABRIC datasets as TNBC or non-TNBC (all other BC subtypes), where samples with missing or incomplete hormone receptor data were removed from analysis. The breakdown of normal, TNBC and non-TNBC for each dataset is shown in Figure S1. PAM50 classification was conducted using the R package “genefu” [9].

CNA data and processed gene-level DM beta values data were obtained from UCSC Xena platform [10] and MethHC database [11], respectively. ChIP-seq data were downloaded from CistromeDB [28] as narrow bed files and annotated with the “ChIPseeker” R package using HG38 as reference genome. This database curated and processed a huge collection of ChIP-seq from public datasets. Potential TF binding sites were screened from 2.0 kb upstream to 2.0 kb downstream of transcription start site (TSS).

In addition, multivariate Cox proportional hazard analysis was used to evaluate the relationship between final glycogenes/TFs and overall survival (OS)/relapse-free survival (RFS).

### 2.3. Data Exploration and Candidate Identification

Sample clustering and glycogen gene expression were investigated using hierarchically clustered heatmap plots from the “pheatmap” R package. Potential glycogen candidates were identified according to the four criteria presented in Figure 1A. First, differential expression of glycogenes was performed using “limma” R package, comparing TNBC to non-TNBC and normal. Second, to identify enriched gene sets and pathways, we adopted an ensemble approach implemented in the “EGSEA” R package, which integrates results from several gene set enrichment (GSEA) methods, thereby reducing method-specific bias [29]. Gene sets enriched in both TCGA and METABRIC datasets with FDR < 0.05 were taken as significant. Third, we assessed the predicative power of candidate glycogenes to discriminate between TNBC, non-TNBC and normal samples using generalized linear models. To prevent model overfitting, we generated a gene network based on the enriched gene sets, where genes that are nodes and edges represent being in the same gene set. We then used the constructed network to perform graph regularization using “glmnet” R package, forcing the model to select genes that are biologically related. Finally, only glycogenes with anabolic or catabolic functions were retained.

### 2.4. Regulatory Mechanism Analysis

Spearman correlation coefficient between gene expression and CNA, as well as DM, was used to assess the regulatory effects for each gene, defining strong (≥0.450), moderate (0.300 to 0.449), weak (0.100 to 0.299) and low/no (<0.100) effects, as described previously [30]. Differential correlation of each gene with CNA/DM between TNBC and non-TNBC was calculated using the following formula:

$$\delta Cor=\left|Cor{\left(g,reg\right)}_{TNBC}-Cor{\left(g,reg\right)}_{non\text{-}TNBC}\right|$$

where δCor is the absolute difference in correlation between gene expression and a regulator (CNA/DM), and Cor(g,reg) is the Spearman coefficient in TNBC and non-TNBC. Statistical testing of differential regulation was performed using the non-parametric Mann–Whitney test, where adjusted *p*-values below 0.05 were considered significant.

## 3. Results

### 3.1. Candidate Discovery

To discover candidate glycogenes unique to TNBC, we made use of three public BC gene expression datasets (TCGA, METABRIC Discovery and METABRIC Validation). An initial list of glycogenes was compiled from the Glycangene database (GGDB) (http://acgg.asia/ggdb2/, accessed on 1 March 2017), a manual review of glycosylation-related pathways in KEGG and a comprehensive literature review [31,32,33,34]. In a pre-processing step, genes not measured in all datasets were removed, leaving 345 glycogenes for investigation (Table S1).

**Figure 3:**
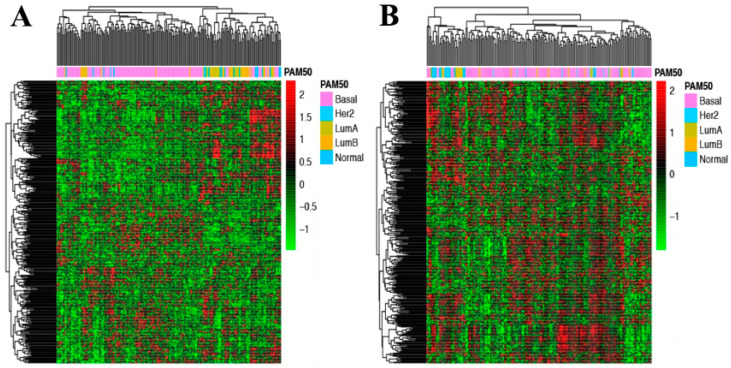
Unsupervised hierarchical clustering of TNBC using 345 glycogene expression. (**A**) Burstein’s dataset (N = 198). (**B**) TCGA dataset (N = 180). PAM50 molecular classification as shown in legend.

We first examined the expression patterns of the 345 glycogenes in TNBC, non-TNBC and normal tissue in each of the three datasets using unsupervised analysis, revealing distinct TNBC glycogene signatures compared to those of non-TNBC and normal in each dataset (Figure S2). Next, we focused on TNBC data, and examined the expression patterns in PAM50 molecular subtypes. As shown in Figure 3, basal-like TNBC tend to cluster in the unsupervised analysis. As basal-like TNBC is the largest subtype of TNBC with relatively homogeneity in terms of tumour development and prognosis [9], we chose to focus on basal-like TNBC for glycogene and glycosylation pathway candidate selection. For simplicity, we will refer to basal-like TNBC as TNBC through this study.

To select TNBC-specific glycogenes, we established four criteria: differential expression in (basal-like) TNBC, present in enriched pathways, predictive of TNBC and of anabolic or catabolic function. 

**Figure 4:**
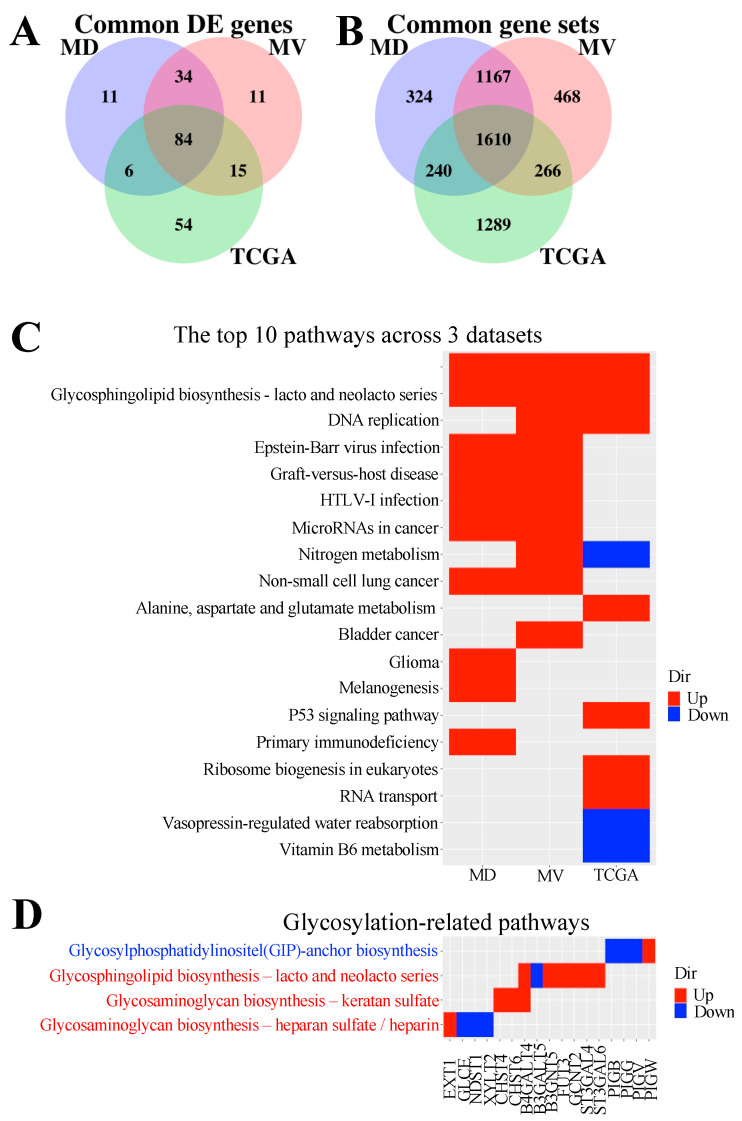
Differentially expressed (DE) genes and pathways for TNBC compared to non-TNBC/normal across three datasets. Venn diagrams showing common DE genes (**A**) and gene sets (**B**). (**C**) The top 10 enriched KEGG pathways across three datasets. (**D**) The common glycosylation-related pathways enriched across all three datasets, with blue font indicating down-regulated pathways, and red font indicating up-regulated pathways.

For criterion one, differential expression was individually analysed in the TCGA, METABRIC Discovery (MD) and Validation (MV) datasets using FDR < 0.05 as criteria. Genes differentially expressed (DE genes) in TNBC compared to normal were first identified. Then DE genes were identified in TNBC compared to normal samples. Only DE genes in both comparisons and regulated in the same direction (up/down) were included in the final list. The analysis returned 159, 135, and 144 DE genes for TCGA, MD and MV, respectively, with 84 DE genes common across all three datasets (Figure 4A). The top 50% of DE genes (42) were selected from this criterion. 

For criterion two, enriched pathways and their regulatory directions were determined by gene set enrichment analysis (GSEA). Enriched pathways that exhibit the same regulatory direction in the comparisons of TNBC to non-TNBC and TNBC to normal with FDA < 0.05 were selected, resulting in 3405, 3341, and 3511 pathways for TCGA, MD and MV datasets, respectively, with 1610 overlapping across all three datasets (Figure 4B). The top 10 significant KEGG pathways derived from GSEA among the three datasets showed only two common pathways, cell cycle and glycosphingolipid biosynthesis lacto/neolacto series (Figure 4C). Discovery of the cell cycle pathway validates our approach as cell cycle deregulation is known in TNBC, and cell cycle-targeting drugs have shown promise in clinical trials [35]. Focusing on glycosylation pathways that were enriched in all three datasets, regardless of ranking, we identified three more pathways: glycosylphosphatidylinositol (GPI)-anchor biosynthesis, which was down-regulated in TNBC, and two pathways in glycosaminoglycan biosynthesis which were up-regulated in TNBC (Figure 4D). Of the 17 glycogenes in these four glycosylation pathways, 13 genes showed concordance of regulation direction with the pathways and were therefore selected as candidates (Figure 4D).

**Figure 5:**
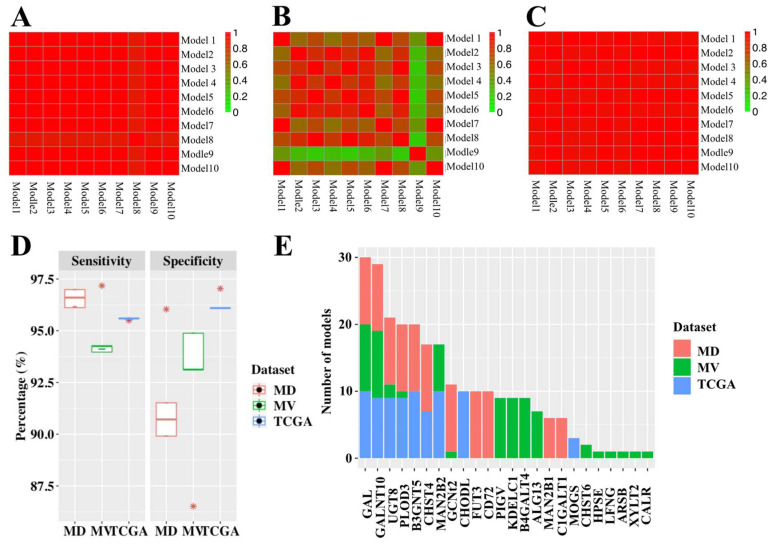
Discovery of TNBC predictive glycogenes. Accuracy and reproducibility of the ten TNBC predictive models for each dataset for (**A**) METABRIC discovery, (**B**) METABRIC validation and (**C**) TCGA. The colour bar indicates the similarity between any two models. (**D**) Distribution of sensitivity and specificity for the predictive models for each dataset. (**E**) Bar chart of frequency of all predictive genes in these 30 models. MD, METABRIC discovery; MV, METABRIC validation. * *p* < 0.05.

For criterion three, predictive glycogenes for TNBC were determined using generalized linear models. Ten models were generated for each dataset with high sensitivity and specificity (Figure 5A–D). The lowest sensitivity of model is in the METABRIC validation dataset, at 94.0%, and the lowest specificity of model is in the METABRIC discovery dataset, at 89.9%. Examination of the predictive genes from all 30 models showed that four genes (GAL, GALNT10, UGT8 and PLOD3) were selected at least once in all datasets, while three additional genes (B3GNT5, CHST4 and MAN2B2) were selected at least once in two datasets (Figure 5E). These seven genes were considered as predictive glycogenes for TNBC.

**Figure 6:**
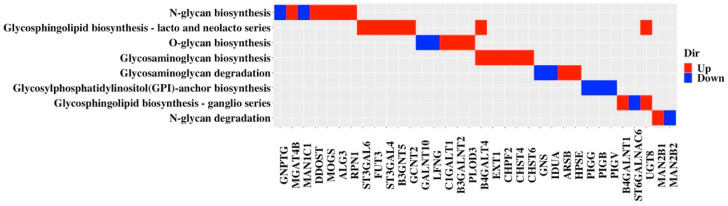
Glycosylation pathways for the 34 candidate TNBC glycogenes. Genes up-regulated in TNBC compared to non-TNBC/normal are labelled as red, while down-regulated genes are labelled as blue.

The shortlisted candidates were finally filtered to retain only anabolic or catabolic genes, to generate a final list of 34 glycogenes (Table S2 and Figure S3) which were grouped to eight functional pathways (Figure 6). Examination of the direction of regulation showed that genes in N-glycan, lacto- and neolacto- series glycosphingolipid and glycosaminoglycan biosynthesis were generally up-regulated, while all three candidate genes involved in GPI-anchor biosynthesis were down-regulated in TNBC (Figure 6). Candidate glycogenes in the other pathways (glycosaminoglycan degradation, N-glycan degradation, ganglio-series glycosphingolipid biosynthesis) were relatively equally up- and down-regulated (Figure 6).

Taken together, our glycogene and pathway discovery identified the lacto-/neolacto- glycosphingolipid biosynthesis pathway to be the top candidate pathway, being one of only two enriched KEGG pathways in all three datasets and containing the highest number (seven) of candidate glycogenes which were all up-regulated in TNBC.

### 3.2. Association of Candidate Glycogenes with Survival in TNBC

Finally, we investigated if the final 34 candidate glycogenes have any association with overall survival (OS) or relapse-free survival (RFS) in TNBC. To address this, we extracted the TCGA data for 97 and 63 TNBC samples with OS and RFS results, respectively. Multivariate Cox analysis with adjustments for age, lymph node metastasis and tumour size, was utilized to evaluate the effect of the final 34 glycogenes on the OS/RFS.

The results indicated a few glycogenes were found to be independent prognostic factors for OS/RFS (Table S3 and Figure S4). Specifically, *EXT1*, *CHST4*, *FUT3*, *PIGV*, *GNPTG*, and *B3GANLT2* were positively associated with OS, while *HPSE* was negatively associated with OS. *CHST4* and *B4GALNT1* were inversely associated with RFS. 

### 3.3. Candidate Glycogene Regulatory Mechanisms

As the expression of several candidate TNBC glycogenes in the same pathways show the same direction of regulation, we hypothesized that they share a common transcriptional regulatory mechanism in TNBC. We addressed this hypothesis by investigating three genetic/transcriptional regulatory mechanisms that are known to be altered in BC, namely copy number alteration (CNA), DNA methylation (DM) and transcription factor (TF) expression. 

**Figure 7:**
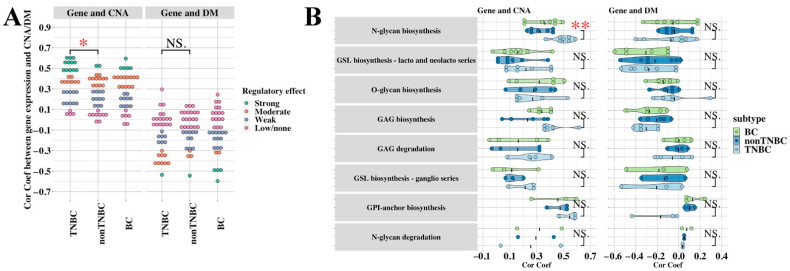
The influence of CNA and DM on candidate glycogene expression in TNBC and non-TNBC. TCGA dataset for gene expression, CNA and DM were analysed for correlation between CNA/DM and gene expression for the 34 candidate glycogenes. Spearman correlation coefficients are shown for all BCs, or divided into TNBC and non-TNBC groups and compared using the Wilcoxon test, at glycogene level (**A**), or grouped into glycosylation pathways (**B**). * *p* < 0.05; ** *p* < 0.001; NS, not significant. GAG, Glycosaminoglycan; GSL, Glycosphingolipid; GPI, Glycosylphosphatidylinositol.

To evaluate the role of CNA and DM in the regulation of candidate glycogene expression in TNBC, we calculated the Spearman correlation coefficient for the 34 candidate glycogenes for CNA–gene expression and DM–gene expression in TCGA multi-omic datasets for all BCs, as well as TNBC versus non-TNBC (Figure 7A). Interaction between CNA and DM regulation was investigated in the BC dataset by plotting the correlation coefficients, which showed that most genes were regulated by either CNA or DM with little interaction (Figure S5). 

As DM is expected to silence gene expression, a gene regulated by DM should have a negative correlation between expression and DM data. Using absolute correlations of 0.45, 0.3 and 0.1 as cut-offs for strong, moderate, weak and low/no regulation, respectively, we show that CNA regulates a larger proportion of the candidate glycogenes compared to DM (Figure 7A). Furthermore, gene–CNA correlation was significantly stronger (non-parametric Mann–Whitney, *p* < 0.05) in TNBC compared to non-TNBC, indicating that candidate genes were more likely to be regulated by CNA in TNBC (Figure 7A). A similar differential regulation was not observed in DM. The top 10 glycogenes showing strong differential regulation by CNA and DM are shown in Table S4 and Figure S6.

Finally, to assess whether differential regulation was pathway specific, we statically tested candidates in each pathway separately. Results show that only N-glycan biosynthesis pathway was differentially regulated by DNA in TNBC (Figure 7B).

### 3.4. Discovery of TFs That Regulate Candidate Glycogenes in TNBC

As the top candidate pathway, lacto-/neolacto- glycosphingolipid biosynthesis, was not differentially regulated by CNA or DM in TNBC, we next investigated a potential role of TF-glycogene regulation in TNBC. We modified a previously described two-step workflow [36] to identify TF-glycogene regulatory effects. Briefly, we used ChIP-seq data from CistromeDB to establish experimentally validated TFs for each glycogene. We then applied feature selection using LASSO-constrained models, including CNA and DM to account for them as possible regulators. Since LASSO models can be unstable, feature selection was repeated 100 times, and TFs were filtered by selection frequency (Figure S7A), optimizing the number of selected TFs by their predictive performance as described previously [36] (Figure S7B).

**Figure 8:**
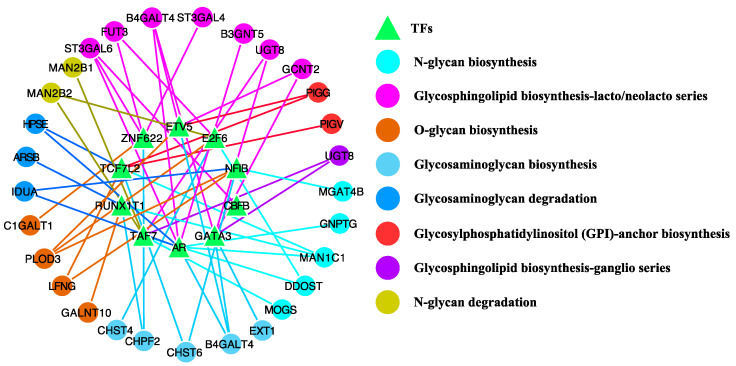
The TNBC-specific network for candidate TFs and glycogenes. The colour keys indicate the glycosylation pathways for the glycogenes.

We then generated two TF-glycogene networks for TNBC and non-TNBC, where nodes in the bipartite graph are TFs and glycogenes, and edges represent the connections selected by the workflow. Since each of the two networks model TF regulation in each group, we performed differential network analysis by subtracting the non-TNBC network from the TNBC network, resulting in a TNBC-specific network with 508 TFs and 864 connections (Table S5). To focus on the master regulators from this extensive list, we retrieved the 10 TFs that interact with four or more candidate glycogenes (Table 1) and visualised their target genes by glycosylation pathway (Figure 8). Intriguingly, androgen receptor (AR) was the most well-connected TNBC-specific TF (Table 1). Of the seven candidate glycogenes regulated by AR, three genes (namely B3GNT5, ST3GAL6 and B4GALT4) are in the glycosphingolipid biosynthesis–lacto/neolacto series pathway (Figure 8). Given that AR antagonists are already actively being investigated as a potential therapy in a subset of TNBCs [37], our findings suggest a potential novel mode of action for AR and AR antagonists, through modulation of glycosylation and GSL function. In addition to AR, ZNF622 and GATA3 represent potentially unexplored therapeutic targets in TNBC, with each TF regulating three TNBC-specific glycogenes in the lacto-/neolacto- glycosphingolipid biosynthesis pathway: ZNF622 (ST3GAL6, FUT3 and ST3GAL4) and GATA3 (GCNT2, UGT8 and B4GALT4).

## 4. Discussion

Despite active development of therapeutics, recent promising TNBC clinical trials only reported several months of additional survival when treated with PARP inhibitors and immunotherapies [17,18]. Therefore, novel approaches and therapeutic targets are urgently needed to address the prognostic disparity for this BC subtype. We focused on glycosylation, which has been implicated in several cancer types, and undertook a bioinformatics approach to analyse glycogene expression data in multiple public transcriptome datasets. By comparing basal-like TNBC with other BCs (here termed non-TNBC), we uncovered 34 glycogenes that are differentially regulated in TNBC. Up-regulation of the lacto-/neolacto- glycosphingolipid (GSL) biosynthesis pathway along with seven candidate glycogenes in this pathway were consistent across three datasets in TNBC. Furthermore, through TF-gene regulatory network analysis, we uncovered three TFs each targeting three glycogenes in this pathway. Interestingly, two of the TFs, AR and GATA3, are being investigated in TNBC [38]. On the other hand, ZNF622 is relatively unexplored. While recent studies have begun to reveal the mechanisms of GSLs in breast cancer [39], this is the first report of the up-regulation of the lacto-/neolacto- GSL pathway specifically in TNBC. Additional studies are warranted to validate the candidate genes/TFs discovered in the current study.

Glycosphingolipids (GSLs) are comprised of diverse glycans conjugated to a sphingoid or ceramide (lipid). GSLs are the major glycoconjugates on the surface of animal cells, where they mediate cell–cell and cell–environment interactions, and modulate signalling through regulation of membrane microdomains and membrane-associated proteins [40]. GSLs are classified by their core glycans, which also align with their biosynthetic pathway into three series/pathways: ganglio-, globo- and lacto-/neolacto- [41]. Alteration in GSL glycosylation is associated with stem cell differentiation [42] and contributes to multiple cancer hallmarks, such as sustained proliferation as well as promoting tumour cell metastasis [43]. Indeed, some of the lacto-/neolacto- glycosphingolipid pathway genes were previously shown to play a critical role in other cancers. For instance, B3GNT5 is elevated in acute myelocytic leukemia, ovarian cancer and glioblastoma compared to healthy controls [44,45,46]. In TNBC, ST3GAL4 and ST3GAL6 were implicated in hypoxia and nutrient deprivation-induced glycosylation change and aggressiveness in TNBC [47,48,49]. UGT8 was previously reported as a potential oncogene in basal-like BC, where inhibition of UGT8 expression suppresses basal-like BC via regulating the sulfatide-αVβ5 axis [50]. These previous functional studies provide support that the elevated lacto-/neolacto- GSL pathway and glycogenes in TNBC likely contribute to the aggressiveness of this subtype. 

To enable down-regulation of the lacto-/neolacto- GSL pathway, we applied an integrative bioinformatics approach to investigate CNA, DM and TF in regulating the list of candidate TNBC glycogenes. This approach revealed three potential master TFs that each target three candidate TNBC-specific glycogenes in this pathway. AR and GATA3 have been previously investigated in TNBC, primarily in non-BL subtypes, while ZNF622 is relatively unknown. None of these TFs have been previously linked to lacto-/neolacto- GSL biosynthesis.

AR is a steroid hormone receptor that acts as a TF in diverse biological functions. Interestingly, depending on which different signalling pathways are activated, AR can promote or suppress proliferation and apoptosis in tumours [51,52]. Different studies have reported AR expression in a diverse proportion of TNBCs from 6% to 75%, with contradictory findings on prognostic implications and mechanisms [53]. While some studies suggested AR overexpression predicted worse prognosis in TNBC [54,55,56], other studies reported elevated AR was associated with a more favourable prognosis in TNBC [57,58], or showed no association between TNBC prognosis and AR expression [59,60]. A clinical trial using the androgen antagonist bicalutamide indicated inhibition of AR has a positive clinical effect on TNBC [61]. Furthermore, AR expression has been correlated with low pathological complete response rate in TNBC, potentially associated with reduced radio sensitivity [38]. The reported discrepancies may be partially due to the molecular heterogeneity of TNBCs, which were generally not pre-selected in these studies. In particular, the luminal apocrine (LAR) subtype of TNBC is characterized by AR expression and has a better prognosis compared to other TNBC subtypes [3,62]. LAR mainly encompasses non-BL subtypes in TNBC, such as HER2 and luminal A subtypes, and thus were not considered in our study as we only included BL TNBC. Future focused dissections of AR in GSL pathway regulation in TNBC subtypes is warranted, with potential for precision repurposing of androgen antagonists as targeted therapy in a subset of TNBC. 

GATA3 transcription factor has previously been associated with less aggressive ER+ BC [63], but recent studies reported the expression of GATA3 in a subset of TNBC, with a broad range of 31.4% to 83% of TNBCs reported in different studies [64,65,66]. Interestingly, GATA3 expression appears to correlate with TNBC metastasis [67], and GATA3 gene variants were detected in 11% of metastatic BCs in a 115 patient cohort [68]. However, there has been no established link between GATA3 expression and glycosylation regulation or GSL pathway. 

ZNF622 (zinc finger protein 622), also known as ZPR9 (zinc finger protein 9), enhances the transcriptional activity of the MYBL2 (also known as B-MYB) transcription factor to regulate cellular growth of neuroblastoma cells [69], but may also have cytoplasmic roles in the regulation of apoptosis [70]. Recent gene expression analyses studies suggest a role for MYBL2 in BC [71] and TNBC [72]. While ZFN622 has been identified as a candidate gene for colorectal cancer metastasis [73], there have not been any reports directly linking ZFN622 to BC or TNBC.

The combination of AR and GATA3 targets five out of seven candidate glycogenes in this pathway (*ST3GAL6*, *B3GNT5*, *B4GALT4*, *GCNT2* and *UGT8*), while addition of ZNF622 adds another glycogene (*FUT3*). Our findings suggest that AR and GATA3 are major potential contributors to the up-regulation of the lacto-/neolacto- GSL biosynthesis pathway in (a subset of) BL TNBC. While our TF-glycogene analysis utilised the experimental ChIP-seq datasets contained in CistromeDB, none of the experiments were conducted in TNBC cells. A literature review focusing on AR, GATA3 and ZNF622 regulation of glycogenes identified a single paper reporting AR binding to a B3GNT5 promoter, using semi-quantitative PCR with ChIP in prostate cancer cell line LAPC-4 [74]. Therefore, this novel pathway and the other candidate glycogenes should be validated in future studies.

## 5. Conclusions

In summary, the current bioinformatics study revealed a list of candidate glycogenes for TNBC. In particular, seven glycogenes of the lacto-/neolacto- GSL biosynthesis pathway and three TFs were identified for therapeutic targeting. With existing AR antagonists such as biclutamide already in trials for TNBC, the discovered glycogenes may be useful for precision medicine in selecting patients, and for the development of novel therapies.

## Figures and Tables

**Table 1:** Candidate transcription factors (TFs) with corresponding target-TNBC-specific glycogenes.

TFs	Target Glycogenes	Number of Targets
AR	*ST3GAL6*, *HPSE*, *IDUA, MAN1C1*, *B3GNT5*, *GNPTG*, *B4GALT4*	7
TCF7L2	*HPSE*, *LFNG*, *CHST6*, *PIGG*, *PIGV*, *MAN1C1*	6
NFIB	*MGAT4B*, *IDUA*, *LFNG*, *CHST6*, *PLOD3*	5
TAF7	*DDOST*, *MAN2B2*, *MAN2B1*, *UGT8*, *MOGS*	5
ZNF622	*ST3GAL6*, *FUT3*, *CHPF2*, *C1GALT1*, *ST3GAL4*	5
E2F6	*CHST4*, *DDOST*, *MAN2B2*, *FUT3*, *PLOD3*	5
GATA3	*EXT1*, *GCNT2*, *UGT8*, *B4GALT4*	4
RUNX1T1	*ARSB*, *CHPF2*, *GALNT10*, *MAN1C1*	4
ETV5	*GCNT2*, *PIGG*, *B4GALT4*, *PLOD3*	4
CBFB	*ST3GAL6*, *IDUA*, *CHST6*, *UGT8*	4

## Data Availability

The data presented in this study are available in Supplementary Materials.

## Supplementary Materials

The following are available online at https://www.mdpi.com/article/10.3390/cancers13133330/s1, Figure S1: The sample distribution in TCGA, METABRIC discovery and validation datasets. Figure S2: Heatmap of all samples in the TCGA and METARIC datasets using the 345 glycogenes. Figure S3: The expression of 34 glycogenes among non-TNBC, TNBC, and normal. Figure S4: Multivariate Cox analysis for OS and RFS within TNBC utilizing 34 glycogenes in TCGA database. Figure S5: Comparison of correlation coefficient between gene and CNA/DM for strongly, moderately and weakly regulated genes. Figure S6: The top 10 differential CNA/DM-regulated glycogenes between TNBC and non-TNBC. Figure S7: Two representative steps for TF selection. (A) The matrix of TFs with different frequency across 100 various λ values for one critical glycan gene. ✓ represents this TF presents in the corresponding model and ✖ represents the TF misses in the corresponding model. (B) A representative example of LASSO-based MSE across all the Freq. The minimal mean MSE corresponds the optimized TF frequency. Table S1: The list of 345 glycogenes. Table S2: The distribution of 34 TNBC-specific genes in KEGG and functional pathways. Table S3: Multivariate Cox analysis for overall survival and relapse-free survival within TNBC samples from TCGA dataset. Table S4: The top 10 genes with most differential correlation between gene and CNA/DM. Table S5: TNBC-specific TF network.
