# Supplementary material for: Differential Regulation of Lacto-/Neolacto- Glycosphingolipid Biosynthesis Pathway Reveals Transcription Factors as Potential Candidates in Triple-Negative Breast Cancer
Source: Cancers (Basel). 2021 Jul 2;13(13):3330. doi: 10.3390/cancers13133330 (PMC8268693; doi:10.3390/cancers13133330)
Supplement: Supplementary file 1 [file cancers-13-03330-s001.zip › Revised supplemental files/Zeng Supplemental Figures Revised.pdf]

Supplemental information for

Differential regulation of lacto-/neolacto-glycosphingolipid biosynthesis pathway reveals transcription factors as potential candidates in triple negative breast cancer

Ruichao Zeng[1], Ahmed Mohamed[1], Kum Kum Khanna[2] and Michelle M. Hill[1,*]

In this document:

Figure S1. The sample distribution in TCGA, METABRIC discovery and validation datasets.

Figure S2. Heatmap of all samples in the TCGA and METARIC datasets using the 345 glycogenes.

Figure S3. The expression of 34 glycogenes among non-TNBC, TNBC, and normal.

Figure S4. Multivariate Cox analysis for OS and RFS within TNBC utilizing 34 glycogenes in TCGA database.

Figure S5. Comparison of correlation coefficient between gene and CNA/DM for strongly, moderately and weakly regulated genes.

Figure S6. The top 10 differential CNA/DM-regulated glycogenes between TNBC and non-TNBC.

Figure S7. Two representative steps for TFs selection.

In Excel file:

Table S1. The list of 345 glycogenes.

Table S2. The distribution of 34 TNBC-specific glycogenes in KEGG and functional pathways

Table S3: Multivariate Cox analysis for OS and RFS within TNBC samples from TCGA dataset.

Table S4. The top 10 glycogenes with most differential correlation between glycogenes and CNA/DM.

Table S5. TNBC-specific TF network.

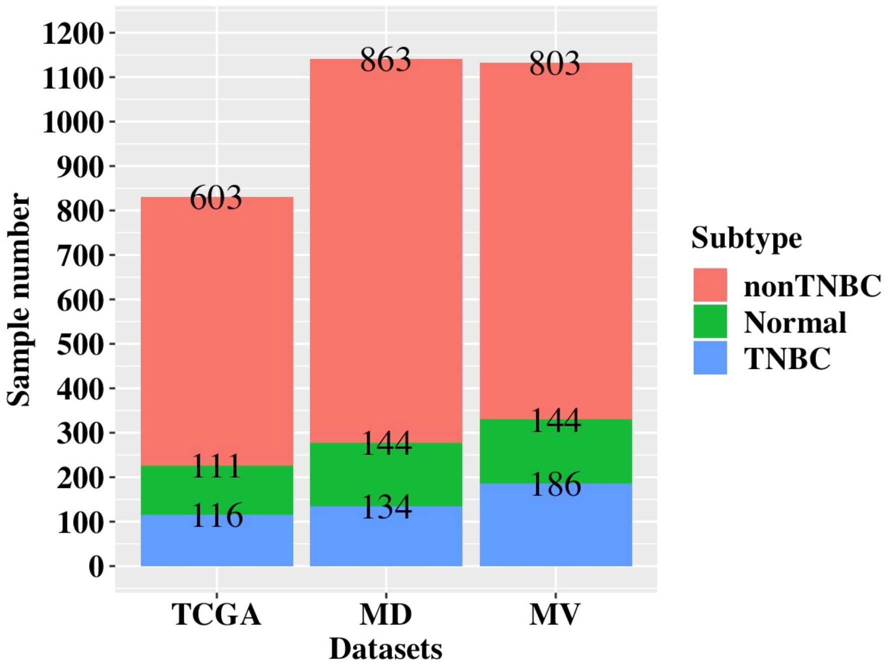

Figure S1. The sample distribution in TCGA, METABRIC discovery and validation datasets.

MD: METABRIC discovery; MV: METABRIC validation.

TCGA

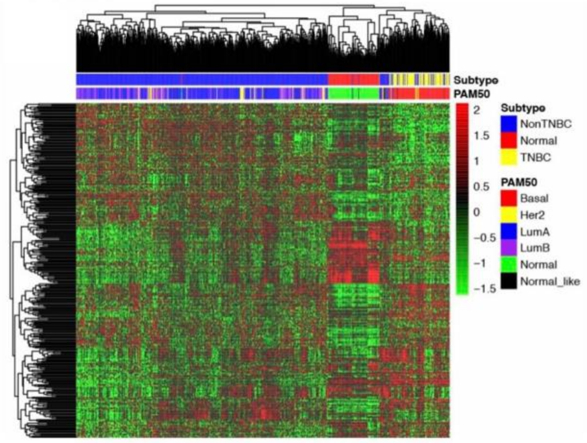

METABRIC Discovery

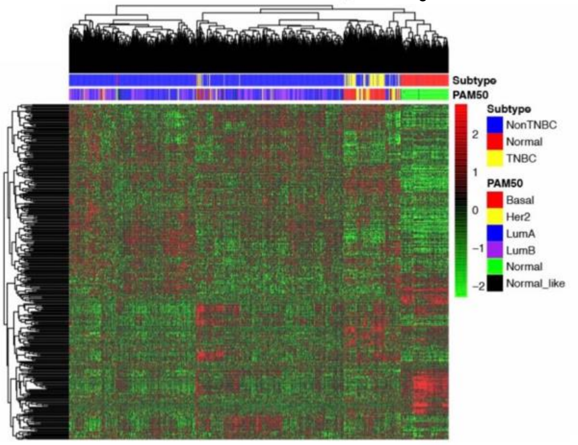

METABRIC Validation

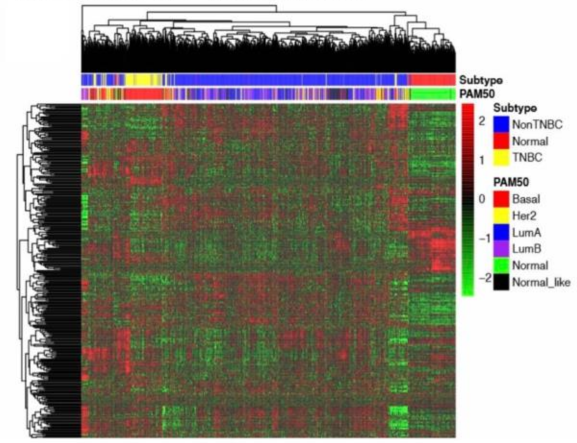

Figure S2. Heatmap of all samples in the TCGA and METARIC datasets using the 345 glycogenes. The legend in the right-side of heatmap shows the different classification subtypes with various colour labels and UNS/UNC indicates unclassified samples.

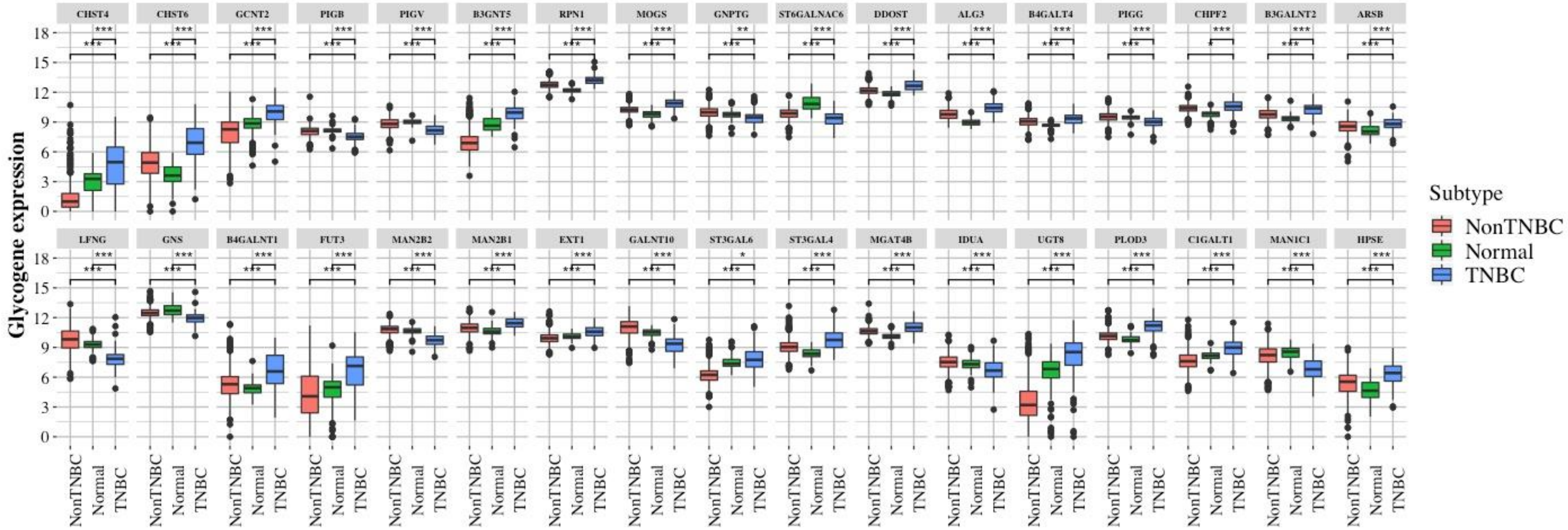

Figure S3. The expression of 34 glycogenes among non-TNBC, TNBC, and normal. * < 0.05; ** < 0.01; * < 0.001.**

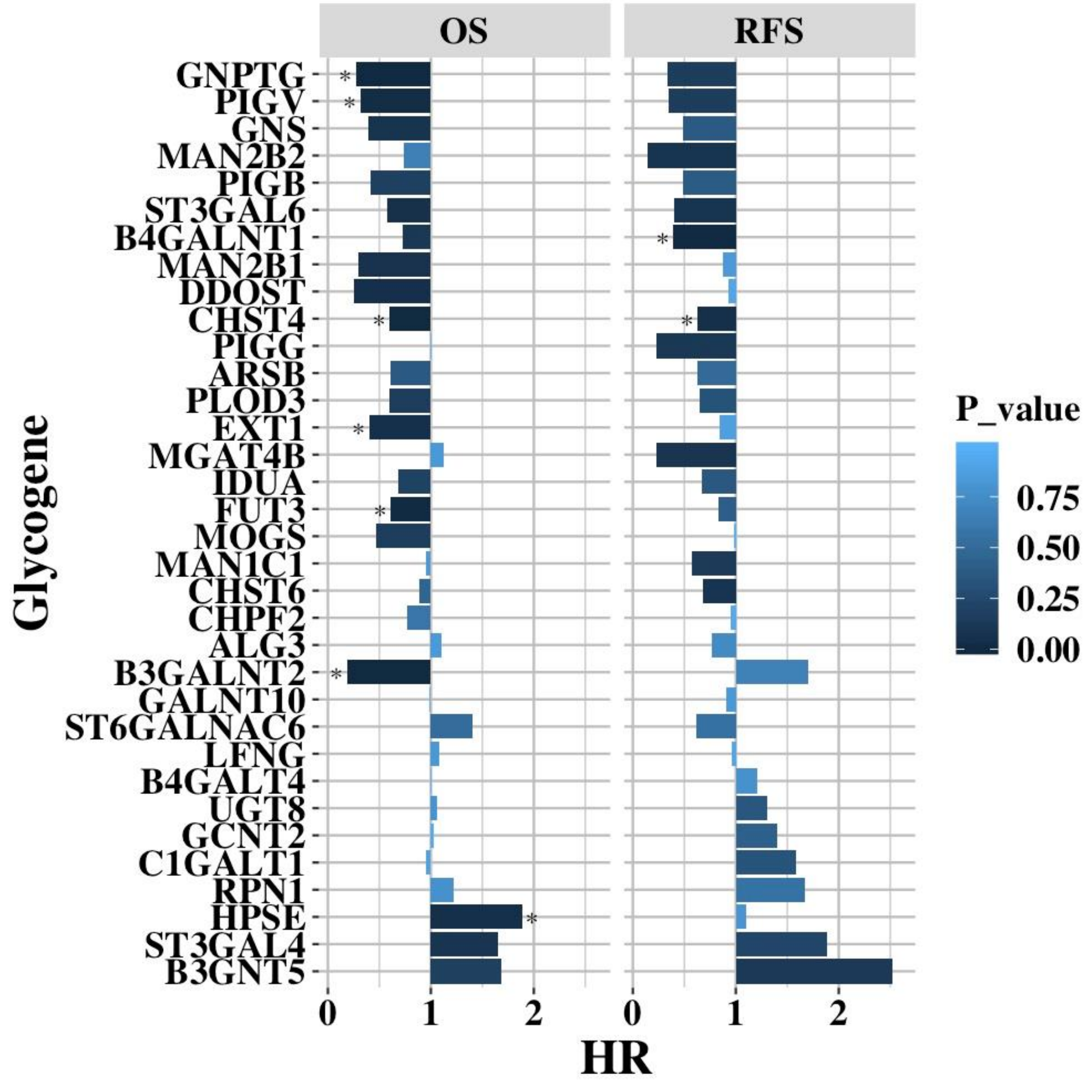

Figure S4. Multivariate Cox analysis for OS and RFS within TNBC utilizing 34 glycogenes in TCGA database. HR, hazard ratio; OS, overall survival; RFS, relapse-free survival.

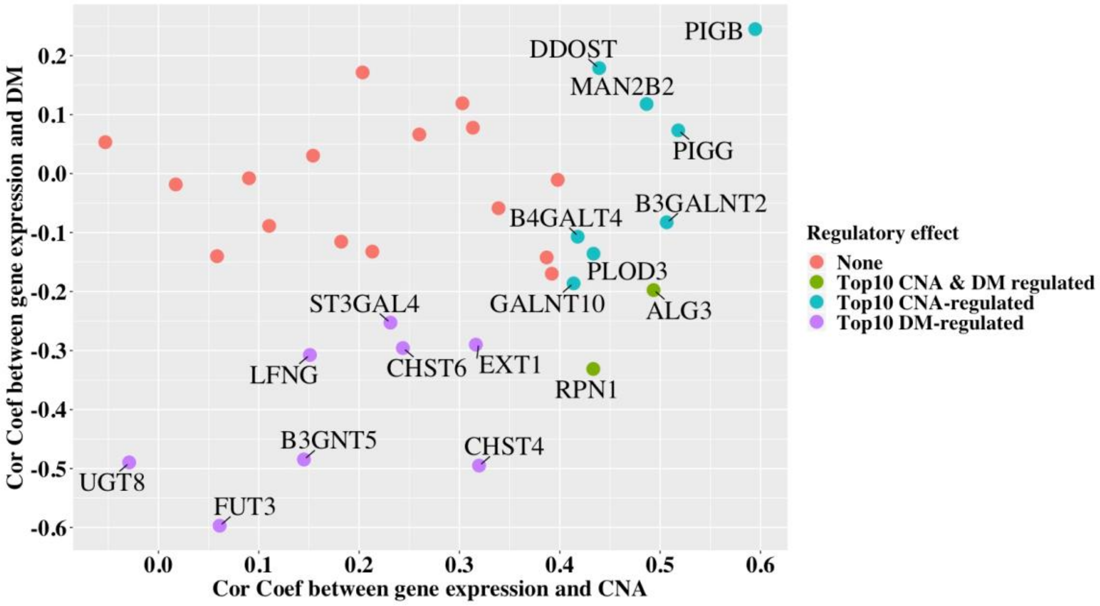

Figure S5. Comparison of correlation coefficient between gene and CNA/DM for strongly, moderately and weakly regulated genes. Orange colour dots: genes other than the top 10 strongest correlating ones to CNA/DM; green colour dots: genes with the top-10 coefficients with both CNA and DM; skyblue colour dots: genes with the top-10 coefficients with CNA; purple colour dots: genes with the top-10 coefficients with DM.

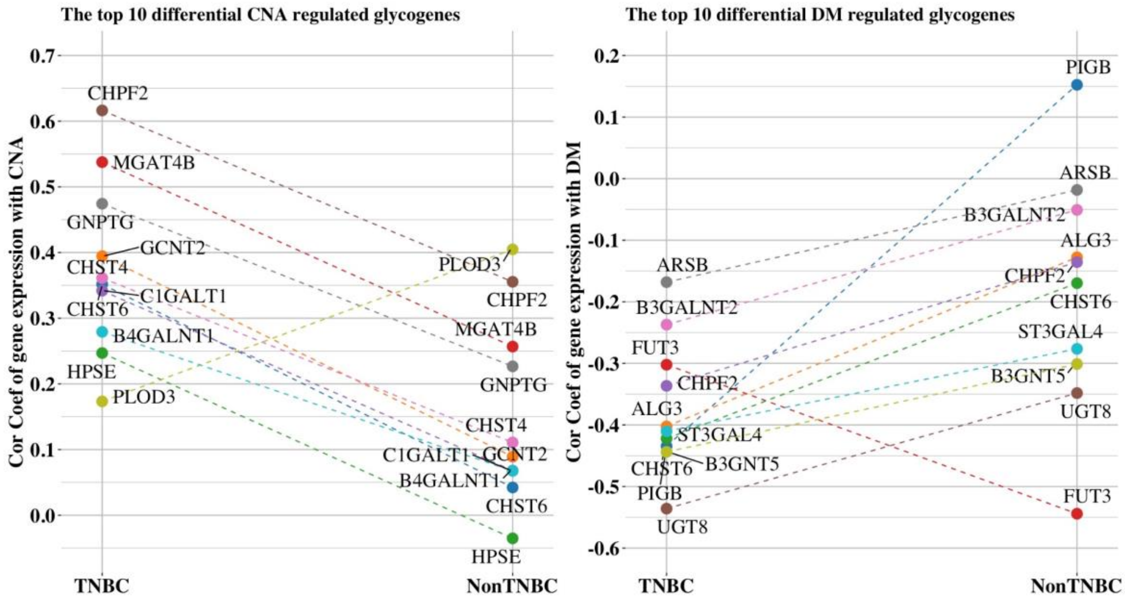

Figure S6. The top 10 differential CNA/DM-regulated glycogenes between TNBC and non-TNBC.

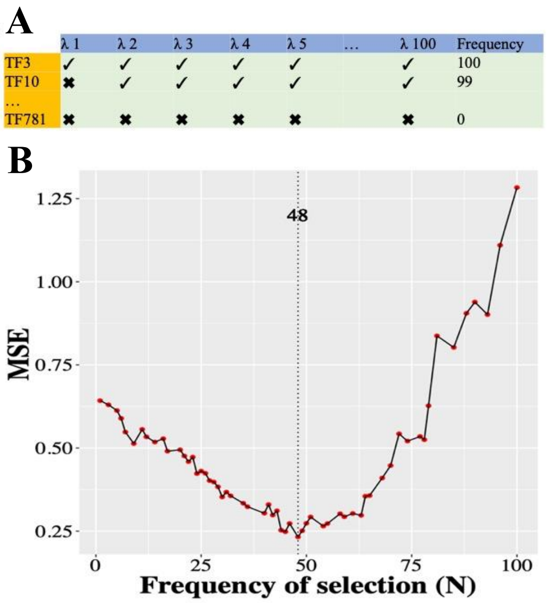

Figure S7. Two representative steps for TFs selection. (A) The matrix of TFs with different frequency across 100 various λ values for one critical glycan gene. ✓ represents this TF presents in the corresponding model and ✖ represents the TF misses in the corresponding model. (B) A representative example of LASSO-based MSE across all the Freq. The minimal mean MSE corresponds the optimized TF frequency.
